# RPS27L Maintains Nucleolar Homeostasis to Regulate Proliferation and Differentiation of Bovine Skeletal Muscle Satellite Cells

**DOI:** 10.3390/ani16162564

**Published:** 2026-08-17

**Authors:** Haina Xu, Xingcun Zhang, Yifan Zhang, Minghui Zhao, Xiangzi Li, Seongho Choi

**Affiliations:** 1 Engineering Research Center of North-East Cold Region Beef Cattle Science & Technology Innovation, Ministry of Education, Yanbian University, Yanji 133002, China; xuseana@163.com; 2 Department of Animal Science, College of Agriculture, Yanbian University, Yanji 133002, China; 3 Department of Animal Science, Qingdao Agricultural University, Qingdao 266000, China; 13783683146@163.com (X.Z.); zhang0421yifan@163.com (Y.Z.); chaomq@ymail.com (M.Z.); 4 Department of Animal Science, Chungbuk National University, Cheongju 28644, Republic of Korea

**Keywords:** RPS27L, bovine skeletal muscle, nucleolar stress

## Abstract

The in vitro expansion of bovine skeletal muscle satellite cells (SMSCs) is critical for cultured meat production. Here, we investigated the role of ribosomal protein S27-like (RPS27L) in regulating satellite cell proliferation and differentiation. RPS27L knockdown markedly inhibited proliferation and promoted myogenic differentiation, whereas RPS27L overexpression exerted opposite effects. Interestingly, both RPS27L depletion and overexpression activated the p53 signaling pathway. Further analyses revealed that disruption of RPS27L homeostasis, regardless of direction, induced nucleolar stress. These findings suggest that proper RPS27L dosage is required to maintain nucleolar homeostasis and normal satellite cell function. Aberrant RPS27L expression activates the nucleolar stress-p53 axis and alters the balance between proliferation and differentiation in bovine skeletal muscle satellite cells. These findings reveal a non-canonical mechanism of p53 regulation in which disturbance of RPS27L homeostasis, rather than simple loss of RPS27L function, activates the nucleolar stress-p53 axis.

## Simple Summary

In this study, we investigated the function of RPS27L in bovine skeletal muscle satellite cells and its role in regulating the p53 signaling pathway. Our results show that both knockdown and overexpression of RPS27L significantly activate p53 signaling. Mechanistically, this activation is not mediated through the canonical MDM2-dependent pathway but is instead driven by nucleolar stress, as evidenced by nucleolar disruption and activation of stress-associated signaling responses. This study is the first to demonstrate that RPS27L regulates p53 through a nucleolar stress–dependent mechanism, thereby expanding the current understanding of RPS27L biology in muscle stem cells and providing new insights relevant to cultured meat research.

## 1. Introduction

With the rapid growth of the global population and increasing concerns regarding environmental sustainability, animal welfare, and food security, conventional livestock production faces significant challenges in meeting future demands for animal-derived proteins. Cultivated meat, produced through the expansion and differentiation of animal cells in vitro, represents a promising alternative approach that may reduce land and water consumption, minimize greenhouse gas emissions, and avoid animal slaughter. Although the current production cost of cultivated meat remains higher than that of conventional livestock production, this is largely attributed to the immature state of cell culture technologies, including the high cost of growth factors, culture media, and bioprocessing systems. Therefore, improving the proliferation efficiency and differentiation capacity of muscle stem cells is critical for the future commercialization of cultivated meat.

Cell-cultured meat production relies on the efficient expansion and differentiation of skeletal muscle satellite cells, which are a population of resident muscle stem cells located beneath the basal lamina of myofibers [1,2]. Upon activation, satellite cells proliferate, differentiate, and fuse to form multinucleated myotubes, thereby contributing to muscle development and repair [3,4]. This process requires precise coordination between cell cycle progression and myogenic differentiation [5]. Dysregulation of this balance can profoundly impair muscle homeostasis and regenerative capacity [6].

**Figure 1:**
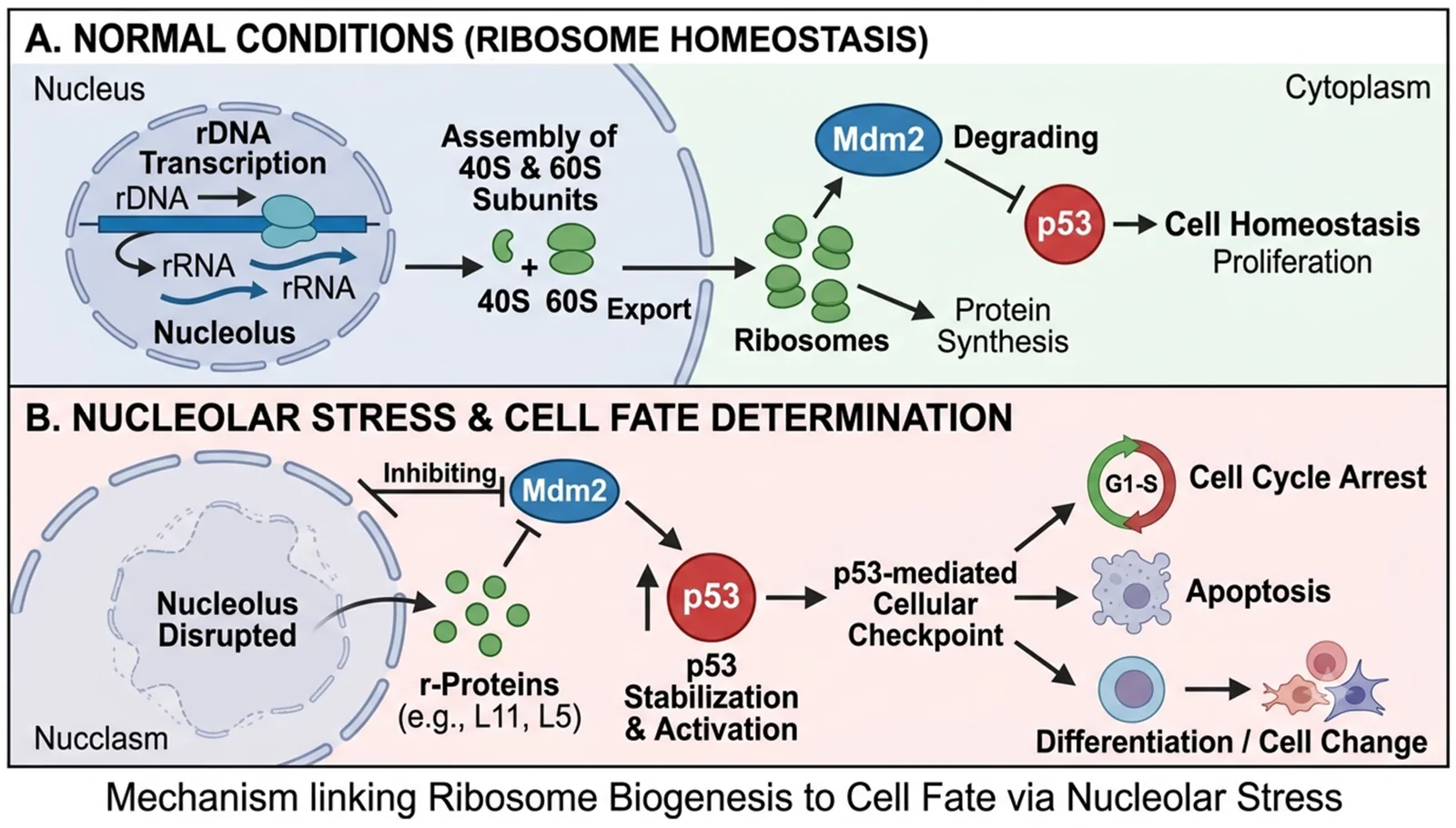
Molecular mechanisms linking ribosomal homeostasis to cell fate regulation. Under physiological conditions, a proper stoichiometric balance of ribosomal proteins (RPs) ensures normal ribosome biogenesis and cell proliferation. Upon perturbation of ribosome biogenesis, nucleolar integrity is disrupted, triggering a nucleolar surveillance mechanism. Free RPs accumulate and inhibit MDM2, leading to the stabilization and activation of the p53 signaling pathway, which ultimately dictates cell cycle regulation, apoptosis, or stem cell differentiation.

Accumulating evidence suggests that ribosome biogenesis and nucleolar integrity are closely linked to stem cell fate determination and tissue development [7,8,9]. Proper stoichiometric balance of ribosomal proteins is essential for ribosome biogenesis and nucleolar integrity [10,11]. Beyond their canonical functions in protein synthesis, ribosomal proteins have emerged as important regulators of cell proliferation, apoptosis, stress responses, and differentiation [12,13,14]. Perturbation of ribosome biogenesis often triggers nucleolar stress, leading to stabilization and activation of p53 signaling pathways [15,16]. This nucleolar surveillance mechanism (Figure 1) serves as a central cellular checkpoint that couples ribosomal homeostasis to cell cycle regulation and cell fate decisions [17].

Ribosomal protein S27-like (RPS27L), a highly conserved homolog of RPS27, has been recognized as an important positive regulator of p53 signaling [18,19,20,21]. Notably, genome annotation of *Bos taurus* has identified an additional RPS27L-like locus (LOC101903478), which is located at a distinct genomic position but encodes a protein sequence identical to RPS27L. Previous studies have demonstrated that RPS27L is a direct transcriptional target of p53 and participates in positive feedback (MDM2 pathway) regulation that stabilizes and sustains p53 activity under cellular stress conditions, [18,21]. In cancer cells, elevated RPS27L expression has been associated with enhanced p53-dependent apoptosis, maintenance of genomic stability, and cellular stress responses [18,19,20,22]. Collectively, these findings support the prevailing view that RPS27L functions primarily as a facilitator of p53-mediated stress signaling. However, whether disruption of RPS27L homeostasis itself can trigger stress-associated p53 activation remains largely unclear. In particular, the biological consequences of RPS27L depletion in skeletal muscle satellite cells have not yet been explored.

Recent evidence suggests that dysregulation of ribosomal protein expression can influence myogenic progression through alterations in nucleolar function, ribosome biogenesis, and translational capacity [7,8,12,14]. Because skeletal muscle differentiation requires extensive biosynthetic remodeling and dynamic regulation of protein synthesis, maintenance of ribosomal homeostasis is likely critical for satellite cell function [3,5,8]. Nevertheless, whether RPS27L participates in the regulation of satellite cell proliferation and differentiation through nucleolar stress-associated pathways remains unknown.

In the present study, we investigated the role of RPS27L in bovine skeletal muscle satellite cells using both gain- and loss-of-function approaches. We found that RPS27L depletion markedly suppressed cell proliferation while promoting premature myogenic differentiation, accompanied by p53 activation and disruption of nucleolar organization. Unexpectedly, excessive RPS27L expression also activated p53 signaling, although it produced distinct effects on myogenic differentiation, suggesting that skeletal muscle satellite cells are highly sensitive to RPS27L dosage imbalance. Collectively, our findings reveal a previously underappreciated role for RPS27L in maintaining nucleolar homeostasis and coordinating the proliferation-to-differentiation transition during myogenesis.

## 2. Materials and Methods

### 2.1. Animal Use and Ethic

The bovine tissue samples used in this study were obtained from one-day-old calves of Yanbian yellow cattle (*Bos taurus*), a native Chinese cattle breed, from a certified commercial slaughterhouse. All sample collection procedures complied with relevant animal welfare guidelines. The experimental protocol was reviewed and approved by the Institutional Animal Care and Use Committee of Yanbian University (Approval No. YD20240510060).

### 2.2. SMSCs Isolation, Culture and Purification

One-day-old calves were selected as the donor source because neonatal bovine SMSCs exhibit high proliferative activity and myogenic potential, facilitating efficient isolation, expansion, and differentiation in vitro. SMSCs were isolated from the gastrocnemius muscles of calves according to previously report [23]. Briefly, the gastrocnemius muscles were excised using sterile scissors and transported to the laboratory within 1 h in ice-cold phosphate-buffered saline (PBS). Under a microscope, the perimysium, blood vessels, nerves, and other trace connective tissues were carefully stripped off with sterile forceps. The remaining muscle tissue was minced and digested with collagenase IV at 37 °C for 5 min. The tissue mixture containing collagenase IV (Gibco, Grand Island, NY, USA) was centrifuged three times at 1200 rpm for 3 min. Subsequently, the resulting cell pellet was resuspended in complete culture medium consisting of Dulbecco’s Modified Eagle Medium (DMEM; Gibco, Grand Island, NY, USA) supplemented with 10% fetal bovine serum (FBS; Gibco, Grand Island, NY, USA) and 1% penicillin/streptomycin (Gibco, Grand Island, NY, USA) and cultured to obtain primary cells in a humidified incubator at 37 °C with 5% CO_2_.

For subculture, primary cells were digested using 0.25% trypsin solution (Gibco, Grand Island, NY, USA). At 4–6 h post-subculture, non-adherent cells were collected and seeded into tissue culture dishes. This differential adhesion method was repeated until passage 3 to enrich the SMSC population.

### 2.3. RNAi and Overexpression

Due to the high sequence similarity between bovine RPS27L and its predicted homolog LOC101903478, the RNA interference strategy used in this study may simultaneously target both transcripts. Therefore, the observed phenotype may represent the combined effect of reducing RPS27L-related transcripts.

For RNAi, three specific siRNA oligonucleotides targeting bovine RPS27L (si-RPS27L-1, si-RPS27L-2, si-RPS27L-3) and a non-targeting negative control siRNA (si-NC) were commercially synthesized (Sangon Biotechnology, Shanghai, China) The transfection of siRNAs was performed using Lipofectamine 3000 RNAiMAX Transfection Reagent (Invitrogen, Carlsbad, CA, USA) following the manufacturer’s protocol. Briefly, bovine SMSCs were seeded in 6-well plates at a density of 2 × 10^5^ cells/well and cultured to 60–70% confluency. For each well, 5 μL of 20 μM siRNA (final concentration: 50 nM) was mixed with 125 μL of Opti-MEM Reduced Serum Medium (Invitrogen) and incubated at room temperature for 5 min. Meanwhile, 5 μL of Lipofectamine 3000 RNAiMAX was diluted in 125 μL of Opti-MEM, incubated for 5 min, and then combined with the siRNA mixture. The resulting complex was incubated for 20 min at room temperature to allow liposome formation, followed by addition to the cell culture medium (without antibiotics). At 48 h post-transfection, total RNA was extracted from the cells, and the knockdown efficiency of each si-RPS27L was evaluated using quantitative real-time reverse transcription PCR (qRT-PCR). The si-RPS27L with the highest knockdown efficiency (≥70% reduction compared to si-NC) was selected for subsequent functional experiments.

The amino acid sequence of human RPS27L is highly conserved with bovine RPS27L, differing by only one conservative amino acid substitution (D/E), suggesting that human RPS27L retains comparable biological activity in bovine SMSCs. For RPS27L overexpression, the full-length coding sequence (CDS) of human RPS27L was synthesized by comercial company (Sangon Biotechnology, Shanghai, China) and cloned into the pcDNA3.1(+) mammalian expression vector to construct the overexpression plasmid pcDNA3.1-RPS27L. The empty pcDNA3.1(+) vector was used as the negative control (pcDNA3.1-NC). Both plasmids were verified by Sanger sequencing (Sangon Biotechnology, Beijing, China) to ensure correct insertion and sequence accuracy.

Transfection of the plasmids was conducted using Lipofectamine 3000 Transfection Reagent (Invitrogen). Bovine SMSCs were seeded in 6-well plates (2 × 10^5^ cells/well) and cultured to 60–70% confluency. For each well, 4 μg of pcDNA3.1-RPS27L or pcDNA3.1-NC was mixed with 125 μL of Opti-MEM, and 10 μL of Lipofectamine 3000 was diluted in another 125 μL of Opti-MEM. The two solutions were combined, incubated for 20 min at room temperature, and then added to the cell culture medium (without antibiotics).

At 48 h post-transfection, total RNA was extracted, and the overexpression efficiency of RPS27L was determined by qRT-PCR (Section 2.5). Cells with a significant overexpression level (≥3-fold increase compared to pcDNA3.1-NC) were used for subsequent proliferation and differentiation assays.

### 2.4. CCK8

Cell proliferation was detected using the Cell Counting Kit-8 (CCK-8; Dojindo Laboratories, Kumamoto, Japan). Briefly, bovine SMSCs from four groups (si-NC, si-RPS27L, pcDNA3.1-NC, pcDNA3.1-RPS27L) were seeded into 96-well plates at 3 × 10^3^ cells/well (5 replicates per group) at 24 h post-transfection. At 0 h, 24 h, 48 h, and 72 h post-seeding, 10 μL CCK-8 reagent was added to each well (1:10 ratio with medium) and incubated at 37 °C for 2 h. The absorbance at 450 nm was measured using a microplate reader (Bio-Rad).

### 2.5. RT-qPCR

Messenger RNA was extracted from different stages of cell differentiation and complementary DNA synthesis were performed as previously described. Briefly, mRNA was extracted from one well of 24-well plate using the commercial Kit followed by routine cDNA synthesis by reverse transcription of RNA using an Oligo(dT) primer and the superscript reverse transcriptase in accordance with the manufacturer’s instructions.

Quantitative reverse trasncription polymerase chain reaction with the primer pairs listed in Table 1 was performed using a Bio-Rad PCR machine (Bio-Rad, Hercules, CA, USA). Relative gene expression was analyzed using the 2^−ΔΔCT^ method. GAPDH mRNA was used as an internal control [24] based on the stability in SMSCs. Three independent experiments were performed with samples in triplicate in each case.

### 2.6. RNA Sequencing and Transcriptome Analysis

Total RNA was extracted from bovine skeletal muscle satellite cells using TRIzol reagent (Invitrogen, Carlsbad, CA, USA) according to the manufacturer’s instructions. RNA concentration and purity were assessed using a NanoDrop spectrophotometer (Thermo Fisher Scientific, Waltham, MA, USA), and RNA integrity was evaluated with an Agilent 2100 Bioanalyzer (Agilent Technologies, Santa Clara, CA, USA). Only RNA samples with high integrity were used for library preparation.

RNA sequencing libraries were constructed and sequenced by OE Biotech (Shanghai, China). Raw sequencing reads were processed to remove adapter sequences, low-quality reads, and reads containing poly-N bases, generating clean reads for subsequent analyses. Clean reads were aligned to the bovine reference genome using HISAT2. Gene-level read counts were generated using HTSeq for downstream differential expression analysis.

Differentially expressed genes (DEGs) between the control and RPS27L knockdown groups were identified using the DESeq2 package (version 1.46.0) in R (version 4.4.1) based on raw count data. Genes with an absolute log2 fold change ≥ 1.0 and an adjusted *p* value < 0.05 were considered significantly differentially expressed.

Functional enrichment analyses, including Gene Ontology (GO), Kyoto Encyclopedia of Genes and Genomes (KEGG), and Gene Set Enrichment Analysis (GSEA), were performed to investigate the biological functions and signaling pathways associated with the DEGs. Heatmaps, volcano plots, principal component analysis (PCA) plots, and enrichment plots were generated using R software packages (version 4.4.1).

### 2.7. Immunofluorescence

Cells were fixed with 3.7% formaldehyde for 30 min at room temperature. After washing with PBS, cells were permeabilized with 0.5% Triton X-100 in PBS for 20 min and blocked with 5% BSA in PBS for 1 h.

Cells were then incubated with anti-NCL primary antibody (1:200, 10556-1-AP, Proteintech, China) at 4 °C overnight. After washing, cells were incubated with Cy3-conjugated Donkey Anti-Rabbit IgG (1:500 dilution, D110052-0100, Sangon, Shanghai, China) for 1 h at room temperature.

Cell nuclei were counterstained with DAPI (C1022, Beyotime Institute of Biotechnology, Shanghai, China) for 20 min. After washing three times with PBS, cells were mounted on glass slides using anti-fade mounting medium containing DABCO.

Representative images were acquired using an immunofluorescence microscope (Nikon Eclipse Ti-U, Tokyo, Japan).

### 2.8. Western Blot

Cells were lysed in SDS lysis buffer supplemented with protease and phosphatase inhibitors. The lysates were collected and heated at 95 °C for 5 min, followed by centrifugation to remove insoluble debris. Protein concentrations were determined using a BCA protein assay kit (P0010, Beyotime Institute of Biotechnology, Shanghai, China).

Equal amounts of protein (20–40 μg) were separated by SDS-PAGE and subsequently transferred onto polyvinylidene fluoride (PVDF) membranes using a wet transfer system for 110 min. After transfer, the membranes were blocked with 5% non-fat milk in TBST for 1 h at room temperature.

The membranes were then incubated overnight at 4 °C with primary antibodies against p53 (1:1000 dilution, 10442-1-AP, Proteintech, Wuhan, China), phospho-p53 (Ser15, 1:1000 dilution, 28961-1-AP, Proteintech, Wuhan, China), and MyHC (1:1000 dilution, 22287-1-AP, Proteintech, Wuhan, China) at the appropriate dilutions. Following three washes with TBST, the membranes were incubated with horseradish peroxidase (HRP)-conjugated secondary antibodies for 1 h at room temperature.

Protein bands were visualized using an enhanced chemiluminescence (ECL) detection system (Bio-Rad Laboratories, Hercules, CA, USA) and captured with a chemiluminescence imaging instrument. Band intensities were quantified using ImageJ (v 1.54, National Institutes of Health, Bethesda, MD, USA) software and normalized to the corresponding loading control.

### 2.9. Data Analysis

All data are presented as the mean ± Standard Error of the Mean (SEM). Statistical analyses were performed using GraphPad Prism 9.0 (GraphPad Software, San Diego, CA, USA). For datasets with 4–6 independent biological replicates, normality was assessed using the Shapiro–Wilk test, and homogeneity of variance was evaluated using the Brown–Forsythe and Bartlett’s tests. Data satisfying the assumptions of normality and homogeneity of variance were analyzed using one-way analysis of variance (one-way ANOVA), followed by Dunnett’s multiple comparisons test for comparisons between each treatment group and the corresponding control group. For datasets with three independent biological replicates (*n* = 3), differences among groups were analyzed using the non-parametric Kruskal–Wallis test. A value of *p* < 0.05 was considered statistically significant, and *p* < 0.01 was considered highly significant.

Heatmaps and transcriptomic data visualization, including PCA plots, volcano plots, and enrichment analyses, were generated using R software packages. RNA-seq differential expression analysis was performed using the DESeq2 package in R.

Quantitative colocalization analysis was conducted using a custom Python (v 3.10) program developed in-house. The software calculates Pearson’s correlation coefficients from fluorescence microscopy images and is provided in DAS.

## 3. Results

### 3.1. RPS27L Is Essential for Bovine SMSCs Proliferation

**Figure 2:**
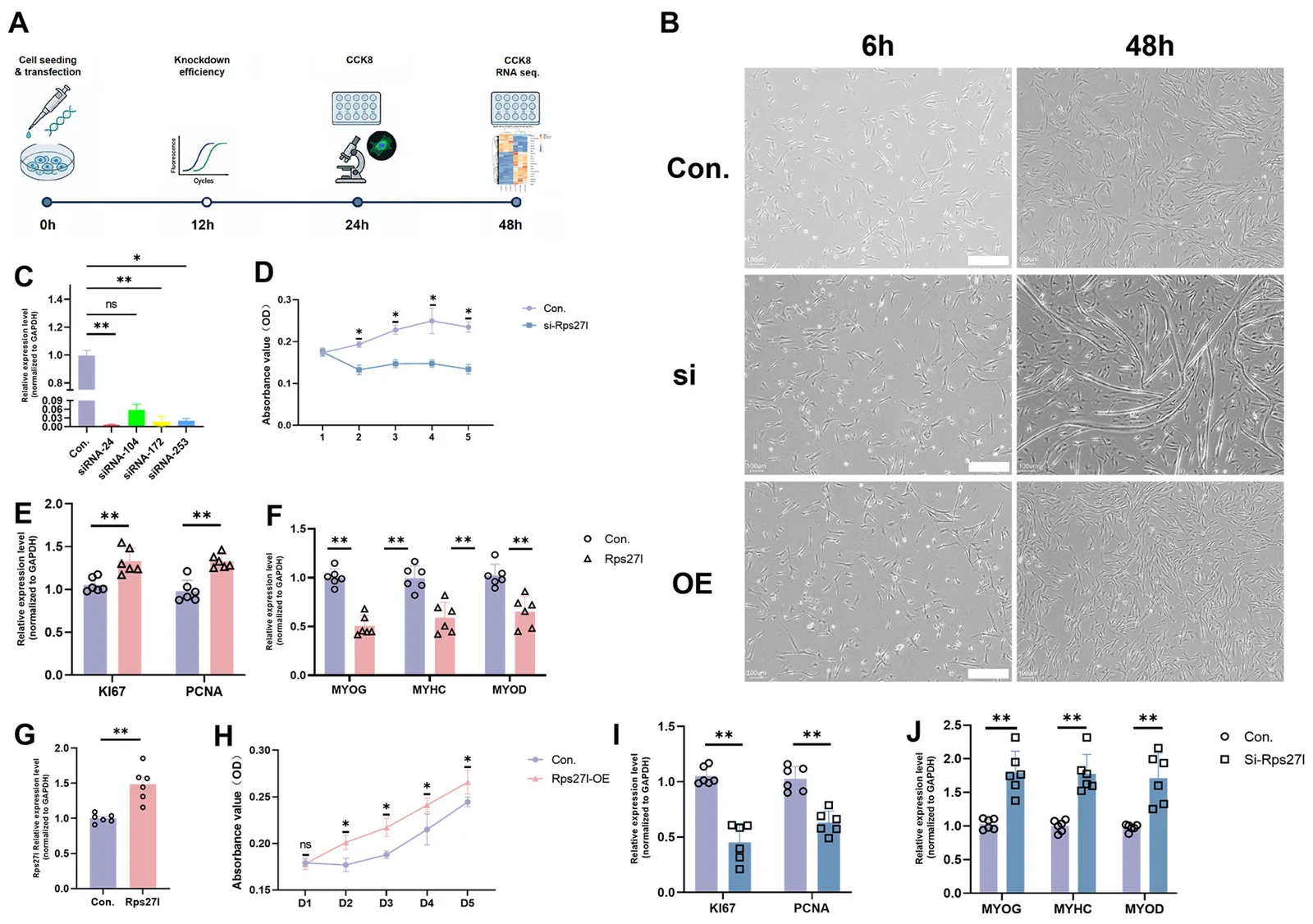
RPS27L is essential for bovine skeletal muscle satellite cell (SMSC) proliferation. (**A**) Schematic diagram of the experimental workflow for RPS27L knockdown and overexpression. (**B**) Morphological changes in bovine SMSCs under different RPS27L expression levels. Scale bar = 200 μm. (**C**) qRT-PCR verification of RNAi knockdown efficiency using four different siRNAs. (**D**) CCK-8 assay showing proliferation curves of SMSCs in control, knockdown (si-RPS27L). (**E**) Expression levels of proliferation markers Ki67 and PCNA following RPS27L knockdown. (**F**) Relative mRNA expression of myogenic differentiation markers (MyoG, MyHC, MyoD) in control and RPS27L knockdown cells. (**G**) qRT-PCR verification of overexpresison of RPS27L. (**H**) CCK-8 assay showing proliferation curves of SMSCs in control, and overexpression of RPS27L. (**I**) Expression levels of proliferation markers Ki67 and PCNA following RPS27L overexpression and (**J**) relative mRNA expression of myogenic differentiation markers (MyoG, MyHC, MyoD) in control and RPS27L overexpression cells. Values represent means ± SEM from at least three separate experiment. Scale Bar = 300 μm. * *p* < 0.05, ** *p* < 0.01, ns, not significant.

To investigate the role of RPS27L in the proliferation of bovine skeletal muscle satellite cells (SMSCs), we performed siRNA-mediated knockdown of RPS27L and subsequently evaluated proliferation- and differentiation-related markers (Figure 2A). A total of four siRNAs targeting RPS27L were screened, and all of them significantly reduced RPS27L mRNA expression levels (Figure 2C). Functional analyses demonstrated that RPS27L depletion almost completely blocked the proliferation of bovine SMSCs (Figure 2B,D). Consistently, the expression levels of proliferation markers Ki67 and PCNA were markedly decreased following RPS27L knockdown (Figure 2E).

**Figure 3:**
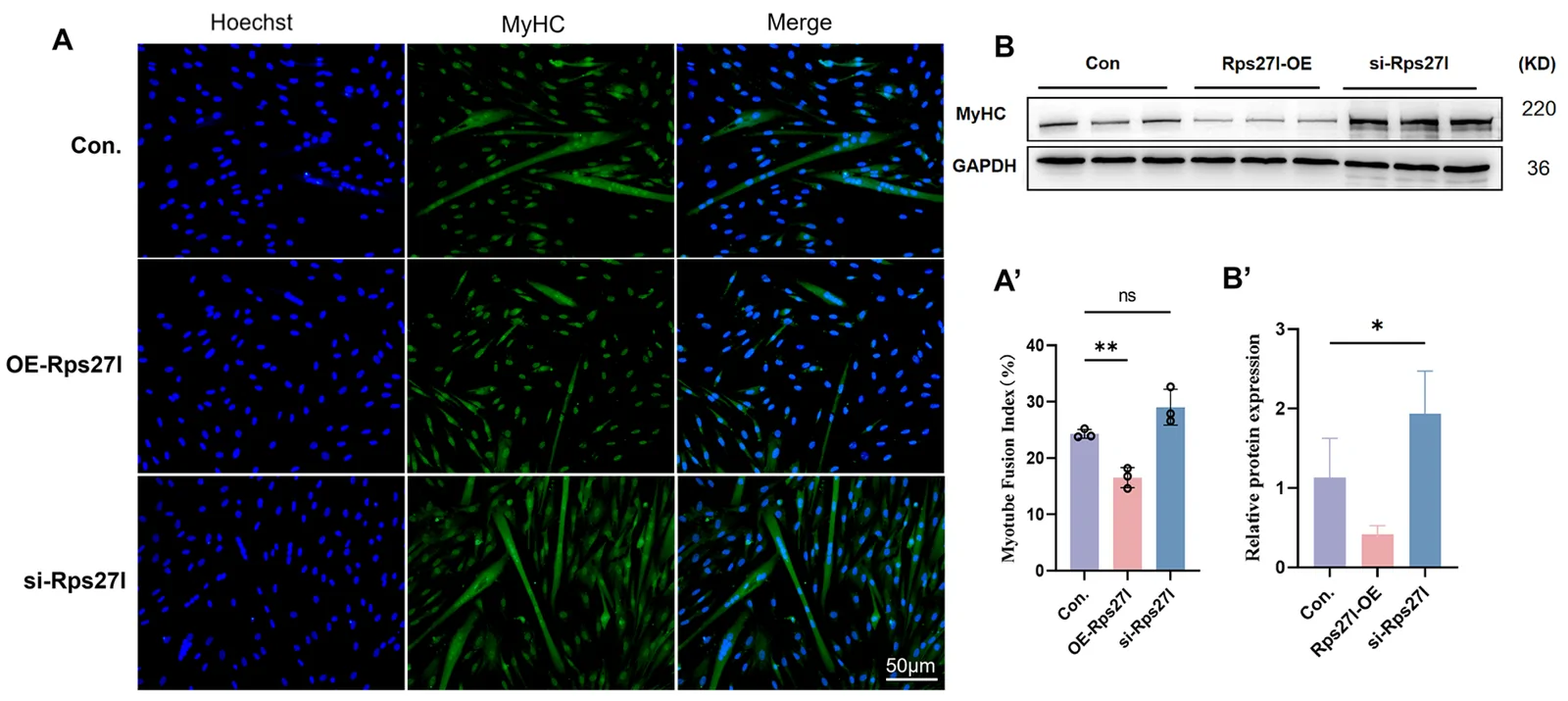
RPS27L modulates myogenic differentiation in bovine SMSCs. (**A**) Immunofluorescence staining of MyHC (red) and Hoechst-stained nuclei (blue) to assess myotube formation. (**A’**) Quantitative analysis of the fusion index across treatment groups. (**B**) Western blot analysis of MyHC protein expression in control, RPS27L overexpression, and RPS27L knockdown cells. (**B’**) Densitometric quantification of MyHC protein levels relative to GAPDH. Values represent means ± SEM from three separate experiment. Scale Bar = 50 μm. * *p* < 0.05, ** *p* < 0.01, ns, not significant.

Interestingly, silencing of RPS27L also resulted in significant upregulation of myogenic differentiation markers, including MyoG, MyHC, and MyoD, in bovine SMSCs (Figure 2F and Figure 3B,B’, *p* < 0.001). Fusion index also increased in knockdown group but decreased in overexpression group (Figure 3A,A’). Differentiation phenotypes were further confirmed by fusion index analysis, immunofluorescence staining, and Western blotting (Figure 3).

### 3.2. Transcriptomic Profiling Reveals That RPS27L Knockdown Suppresses Cell-Cycle-Associated Programs

**Figure 4:**
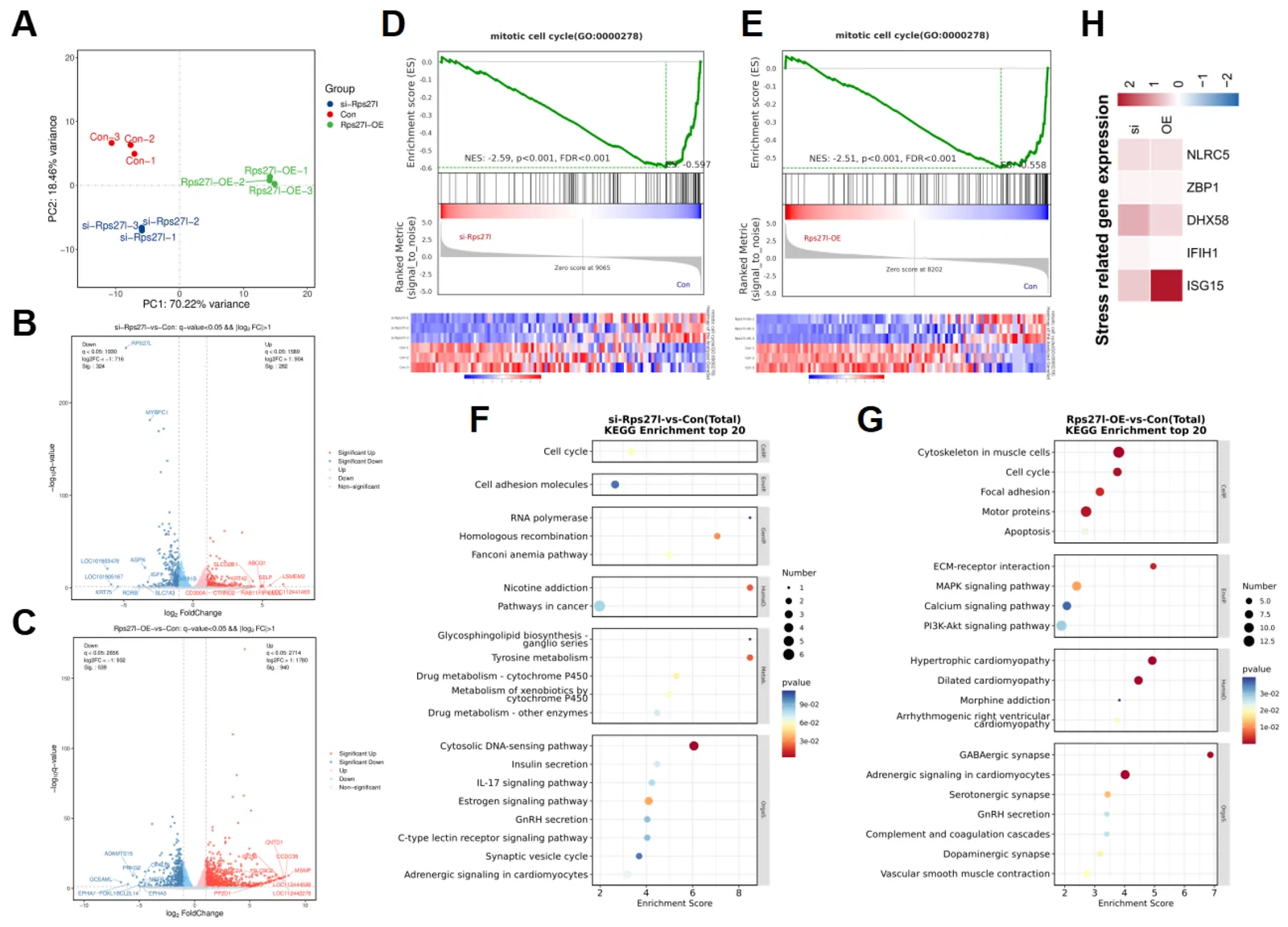
Transcriptomic analysis reveals RPS27L-dependent cell cycle and stress response programs. (**A**) Principal component analysis (PCA) demonstrating transcriptional separation among control, knockdown, and overexpression groups. (**B**,**C**) Volcano plots showing differentially expressed genes (DEGs). (**D**,**E**) GSEA enrichment plots highlighting cell cycle-associated pathways. (**F**,**G**) KEGG enrichment analysis of DEGs in knockdown and overexpression groups. (**H**) Heatmap of stress-associated genes (ISG15, IFIH1, DHX58, ZBP1, NLRC5) upregulated by RPS27L imbalance.

To identify molecular pathways affected by RPS27L perturbation, transcriptomic profiling was performed in control, knockdown, and overexpression SMSCs. PCA demonstrated clear separation among the three groups, indicating substantial transcriptional remodeling following alteration of RPS27L expression (Figure 4A).

Differential expression and enrichment analyses revealed significant changes in pathways associated with cell proliferation (Figure 4B–G).

Notably, several stress-associated genes, including ISG15, IFIH1, DHX58, ZBP1, and NLRC5, exhibited increased expression following both RPS27L depletion and overexpression (Figure 4H), suggesting activation of a common stress-responsive transcriptional program in response to RPS27L imbalance.

### 3.3. RPS27L Imbalance Induces Nucleolar Stress-Associated p53 Activation

**Figure 5:**
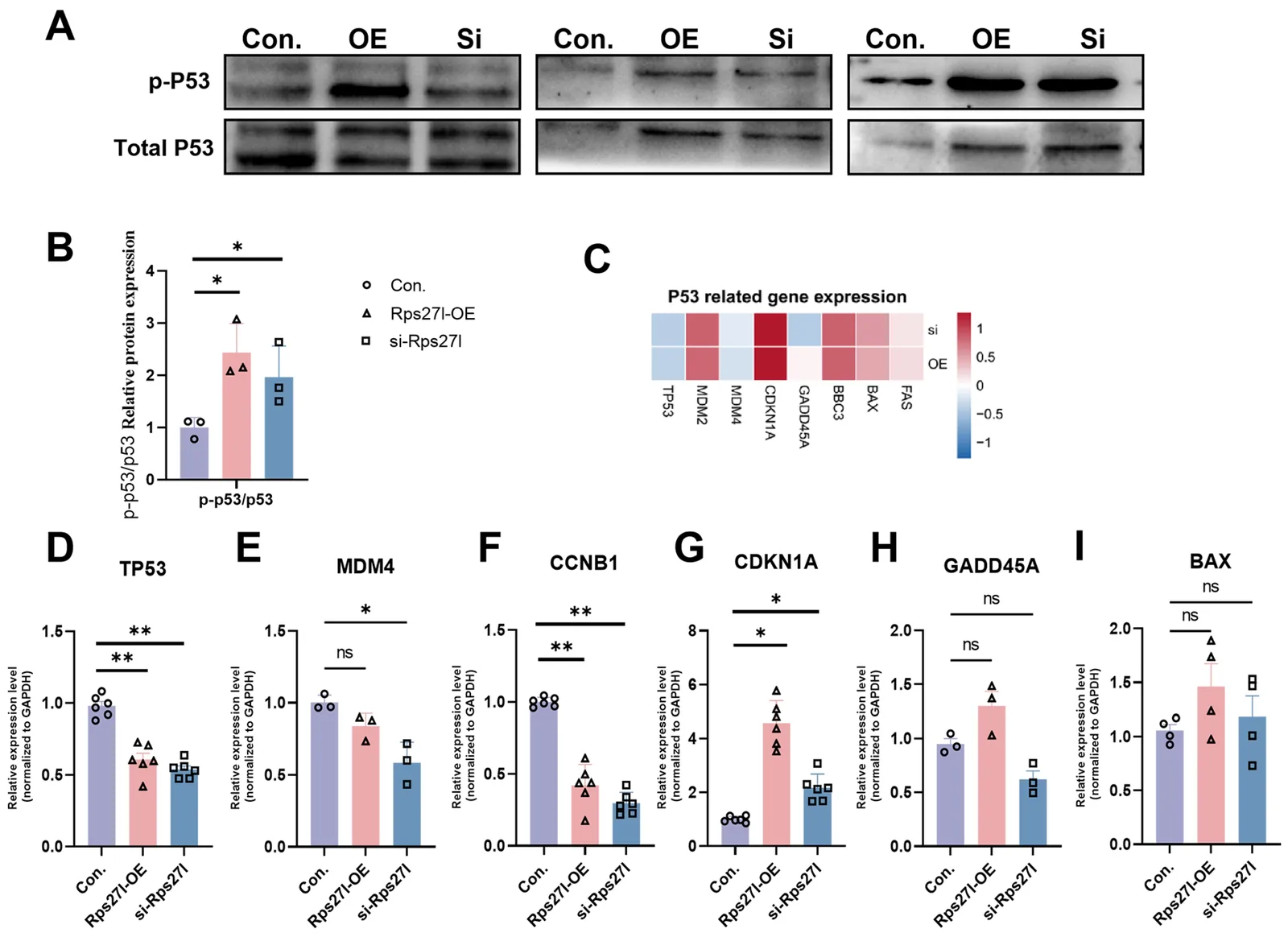
RPS27L imbalance triggers p53 pathway activation. (**A**) Western blot analysis showing phosphorylation of p53 (Ser15) and total p53 levels under RPS27L knockdown and overexpression. (**B**) Quantification of phospho-p53 protein levels. (**C**) Heatmap showing the expression patterns of p53 signaling-related genes. (**D**–**I**) qRT-PCR validation of p53 downstream target genes (TP53, MDM4, CCNB1, CDKN1A, GADD45A and BAX). Values represent means ± SEM from at least three separate experiment. * *p* < 0.05, ** *p* < 0.01, ns, not significant.

The classical regulatory model suggests that RPS27L is required for maintaining p53 activity. However, in the present study, sequencing data revealed that both RPS27L overexpression and knockdown induced similar expression patterns of multiple p53 pathway-associated genes (Figure 5C). The results were confirmed by qRT-PCR (Figure 5D–I). These findings suggest that RPS27L may play an important role in maintaining the homeostatic stability of the p53 signaling pathway.

Interestingly, both depletion and overexpression of RPS27L induced phosphorylation of p53 protein (Figure 5A,B). To further investigate the molecular mechanism underlying p53 activation following RPS27L knockdown, we examined the subcellular localization of nucleolin (NCL), a key nucleolar marker protein, in differently treated cells.

**Figure 6:**
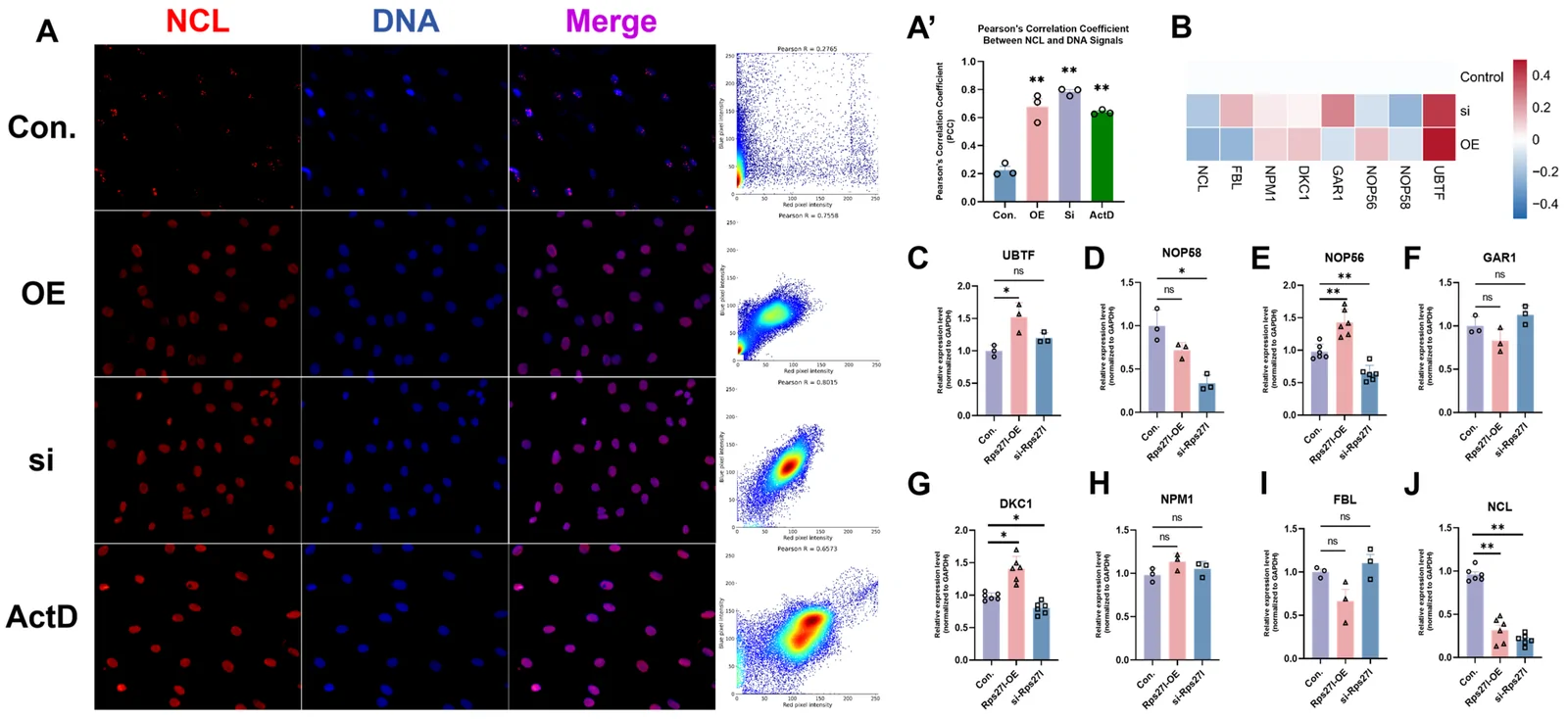
RPS27L homeostasis maintains nucleolar integrity. (**A**) Immunofluorescence imaging of nucleolin (NCL, red) and DAPI (DNA, blue) showing nucleolar redistribution following RPS27L perturbation compared to Actinomycin D (ActD) treatment. (**A’**) Quantification of NCL and DAPI colocalization using Pearson’s correlation coefficient. (**B**) Heatmap showing the relative expression of nucleolar function-associated genes. (**C**–**J**) qRT-PCR quantification of genes involved in nucleolar organization and ribosome biogenesis, including UBTF, NOP56, NOP58, GAR1, DKC1, NPM1, FBL, and NCL. Values represent means ± SEM from at least three separate experiments. Scale Bar = 300 μm. * *p* < 0.05, ** *p* < 0.01, ns, not significant.

Immunofluorescence analysis revealed that RPS27L depletion caused a pronounced redistribution of NCL. In control cells, NCL was predominantly localized within the nucleolar region, whereas after RPS27L knockdown, NCL became diffusely distributed throughout the nucleus (Figure 6). Consistently, Pearson’s correlation coefficient between NCL and DAPI increased from 0.28 to 0.81 after knockdown, indicating severe disruption of nucleolar organization and suggesting activation of nucleolar stress signaling.

**Figure 7:**
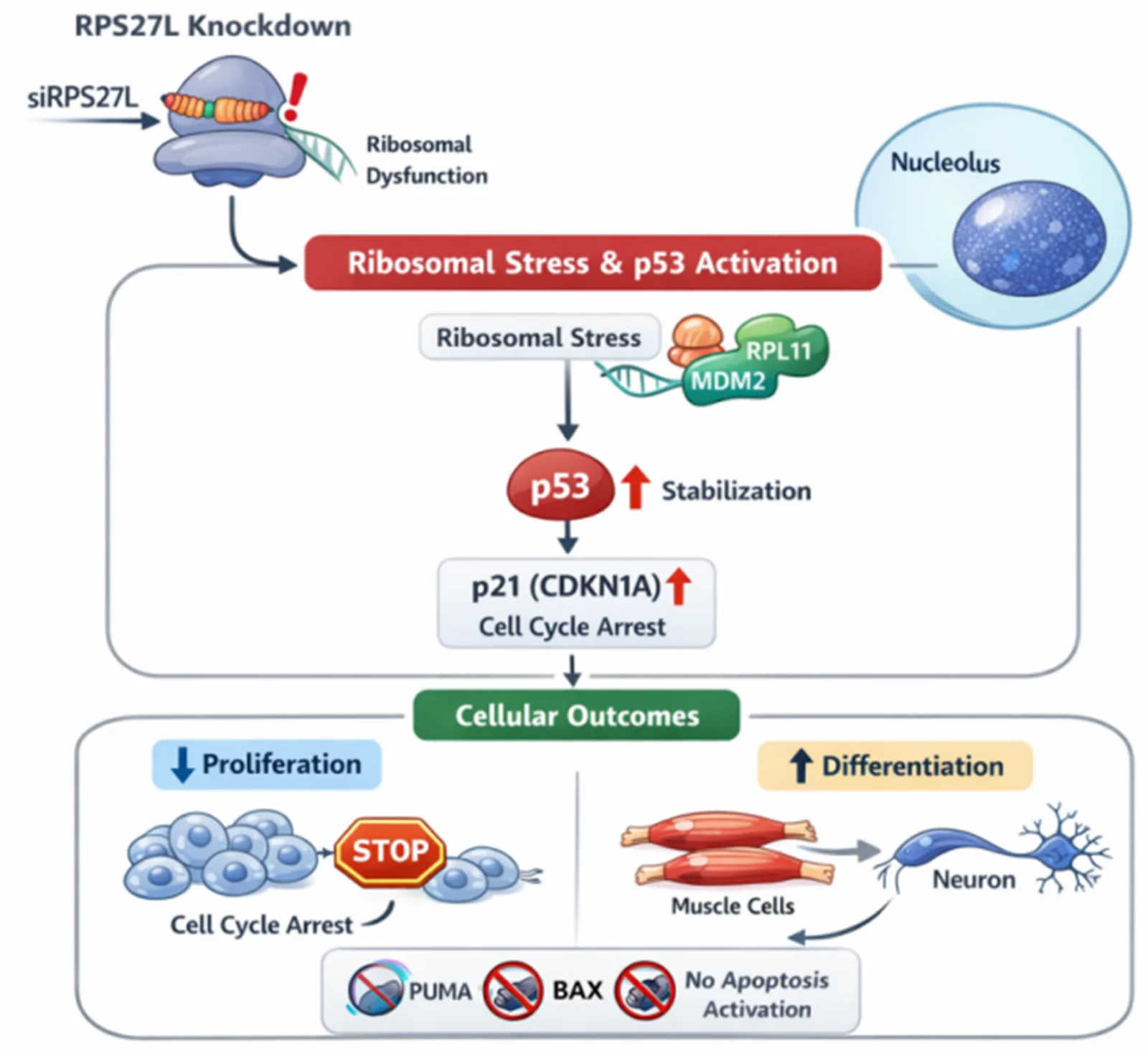
Proposed model of RPS27L-mediated regulation of satellite cell fate. A summary model illustrating how RPS27L dosage imbalance triggers ribosomal stress, resulting in nucleolar dysfunction and p53 signaling activation, which ultimately dictates the proliferation-to-differentiation transition in bovine skeletal muscle satellite cells.

Collectively, these results indicate that RPS27L imbalance induces nucleolar stress and activates p53 signaling, suggesting a coordinated regulatory mechanism governing satellite cell fate (Figure 7).

## 4. Discussion

In the present study, we demonstrated that RPS27L homeostasis plays a critical role in maintaining proliferation-differentiation balance in bovine skeletal muscle satellite cells. Loss of RPS27L markedly suppressed cell proliferation, activated p53 signaling, disrupted nucleolar organization, and promoted myogenic differentiation. Interestingly, RPS27L overexpression also induced p53 activation, although it produced distinct phenotypic outcomes compared with RPS27L depletion. These findings suggest that appropriate RPS27L dosage is essential for maintaining nucleolar homeostasis and normal satellite cell function.

Previous studies have primarily linked RPS27L to p53 regulation through the canonical MDM2-dependent ubiquitination pathway [18,19,25]. RPS27L has been reported to participate in p53-MDM2 regulatory feedback circuits, thereby modulating p53 stability and degradation [18,19]. In this classical model, alterations in RPS27L expression influence p53 activity mainly through changes in ubiquitin-mediated proteasomal turnover. These observations established RPS27L as a downstream effector and modulator of the p53 network in stress responses and tumor biology.

However, the findings of the present study suggest that RPS27L may also regulate p53 through a distinct non-canonical mechanism associated with nucleolar homeostasis [11,16,26]. Several lines of evidence support this interpretation. First, RPS27L depletion caused profound redistribution of nucleolin (NCL) from nucleolar-restricted localization to diffuse nuclear dispersion, indicating disruption of nucleolar integrity [16,27,28]. Second, transcriptomic analysis revealed strong enrichment of cell cycle suppression pathways and stress-associated transcriptional programs. Third, phosphorylation of p53 at Ser15 was significantly elevated following RPS27L depletion. Ser15 phosphorylation is commonly associated with nucleolar stress, replication stress, or DNA damage signaling mediated by ATM/ATR family kinases [29,30], rather than simple stabilization through inhibition of ubiquitin-mediated degradation. Collectively, these observations suggest that loss of RPS27L activates a nucleolar stress surveillance pathway upstream of p53 activation.

Nucleolin (NCL) is a multifunctional nucleolar protein that plays an important role in ribosome biogenesis, nucleolar organization, and stress responses [31]. Previous studies have demonstrated that NCL can directly bind to p53 mRNA and suppress its translation under normal conditions, thereby maintaining low basal p53 activity [32,33]. In skeletal muscle cells, the NCL–p53 regulatory axis has also been implicated in myogenic differentiation, suggesting that dynamic regulation of NCL localization and function is important for controlling muscle cell fate decisions [32]. In the present study, RPS27L depletion induced a marked redistribution of NCL from the nucleolar region to the nucleoplasm, accompanied by p53 Ser15 phosphorylation and activation of p53 downstream targets. These observations suggest that disruption of RPS27L homeostasis may impair nucleolar integrity and alter NCL-mediated regulation of p53 signaling, thereby contributing to the inhibition of proliferation and promotion of myogenic differentiation in bovine satellite cells. However, whether RPS27L directly regulates NCL activity or affects p53 translation through NCL requires further investigation.

Although p53 is widely recognized as an apoptosis-associated tumor suppressor, its activation does not always result in apoptotic cell death [34]. In skeletal muscle cells, p53 has been reported to cooperate with myogenic regulatory factors such as MyoD to promote cell cycle withdrawal and differentiation [32]. Consistent with this concept, our results showed increased p53 activation and CDKN1A/p21 expression following RPS27L depletion, whereas BAX expression remained unchanged, suggesting that RPS27L regulates satellite cell fate through a p53-dependent growth arrest and differentiation pathway rather than apoptosis induction.

In addition to the redistribution of nucleolin (NCL), transcriptomic analysis further supported the presence of nucleolar perturbation following RPS27L imbalance. Several nucleolar function-associated genes exhibited altered expression patterns in both the knockdown and overexpression groups. Notably, UBTF, a key regulator of rRNA transcription and nucleolar organization [35,36], was consistently upregulated, suggesting compensatory activation of ribosome biogenesis-related pathways under conditions of RPS27L imbalance. In contrast, NOP58, an essential component of box C/D snoRNP complexes involved in pre-rRNA processing and ribosome maturation [37], showed a tendency toward downregulation, implying potential defects in nucleolar RNA processing dynamics. In addition, NPM1 expression displayed a mild increase in both groups, which may reflect adaptive responses to nucleolar stress and altered ribosomal assembly homeostasis. Although these transcriptional changes alone are insufficient to conclusively establish nucleolar stress, their combined occurrence together with NCL redistribution and p53 Ser15 phosphorylation strongly supports the involvement of nucleolar stress-associated signaling pathways in response to disturbed RPS27L dosage.

Importantly, we observed that both RPS27L depletion and overexpression induced p53 activation and produced partially overlapping downstream transcriptional responses, including upregulation of CDKN1A, BBC3, FAS, and BAX. This phenomenon is difficult to fully explain solely through the classical MDM2-mediated ubiquitination model. Instead, our data support the possibility that perturbation of RPS27L dosage itself disrupts ribosomal homeostasis and triggers stress signaling independently of conventional p53 degradation pathways. Ribosome biogenesis requires highly coordinated stoichiometric assembly of ribosomal proteins and rRNA. Either insufficient or excessive accumulation of individual ribosomal proteins may impair ribosomal assembly dynamics and activate nucleolar surveillance mechanisms [9,17]. Therefore, our findings expand the current understanding of RPS27L biology by suggesting that RPS27L is not merely a passive regulator of p53 ubiquitination, but may function as an active guardian of nucleolar integrity.

Another important finding of this study is that RPS27L depletion strongly inhibited cell cycle progression while simultaneously promoting myogenic differentiation. RNA-seq analysis revealed significant enrichment of cell cycle-associated pathways, consistent with reduced expression of proliferation markers Ki67 and PCNA. Satellite cell differentiation requires irreversible withdrawal from the cell cycle, and activation of p21-dependent signaling is known to facilitate this transition [1]. The marked induction of CDKN1A observed in the si group likely contributed to proliferation arrest and promoted initiation of the myogenic differentiation program, as reflected by elevated MyoD, MyoG, and MyHC expression [38].

Interestingly, although RPS27L overexpression produced even stronger p53 activation than knockdown, proliferative inhibition was relatively modest, whereas terminal differentiation appeared to be impaired, as evidenced by near absence of MyHC protein expression. This suggests that distinct intensities or contexts of p53 activation may produce different biological outcomes in satellite cells. Mild or adaptive p53 activation may interfere with differentiation-associated transcriptional remodeling without fully inducing irreversible cell cycle arrest, whereas severe nucleolar stress induced by RPS27L depletion may drive cells toward premature differentiation following proliferative shutdown.

Based on these findings, we propose a dosage-sensitive model in which RPS27L maintains satellite cell homeostasis through preservation of nucleolar integrity and balanced p53 activity. Under physiological conditions, appropriate RPS27L expression supports normal ribosome biogenesis and proliferation–differentiation coupling. In contrast, either depletion or excessive accumulation of RPS27L disrupts ribosomal equilibrium, activates nucleolar stress-associated p53 signaling, and alters satellite cell fate decisions.

Collectively, our findings suggest potential involvement of a nucleolar stress-associated mechanism in RPS27L-mediated p53 regulation in bovine skeletal muscle satellite cells. In addition to its reported role in the MDM2-mediated ubiquitination pathway, RPS27L appears to function as an important regulator of nucleolar homeostasis and ribosomal stress responses. These findings broaden the current understanding of RPS27L biology and provide new insights into the relationship between nucleolar integrity, p53 signaling, and skeletal muscle stem cell fate regulation.

## 5. Conclusions

In this study, we systematically investigated the role of RPS27L in bovine skeletal muscle satellite cells, a key cell type for cultured meat production. Our results demonstrate that both depletion and overexpression of RPS27L significantly impair the balance between cell proliferation and myogenic differentiation, indicating that precise dosage control of RPS27L is essential for maintaining normal satellite cell function. Importantly, we show that RPS27L imbalance consistently activates the p53 signaling pathway, highlighting its central role in regulating satellite cell fate decisions under conditions of cellular perturbation.

Mechanistically, we reveal that RPS27L regulates p53 signaling through a nucleolar stress–dependent pathway rather than the canonical MDM2-mediated mechanism. Disruption of RPS27L homeostasis leads to nucleolar dysfunction, which serves as a trigger for p53 activation and subsequent changes in cell cycle and differentiation programs. These findings uncover a previously unrecognized RPS27L–nucleolar stress–p53 regulatory axis and provide new insight into the coordination of ribosomal homeostasis and myogenic progression. This study not only advances our understanding of muscle stem cell biology but also offers potential implications for improving the efficiency of cultured meat production.

## Figures and Tables

**Table 1:** Primers used in the present study for qPCR analysis.

Genes	Primer Sequence	Product Size (bp)	Tm (°C)	Accession Number
*RPS27L*	F:CATCCTTCCTTGGACGAG	147	57	NM_001040578
	R:TACGCAAAGCACTACTGTCTG			XM_059885160.1
*KI67*	F:CCAAAGGCTGTTCTCAA	99	54	XM_015460791
	R:AAGTGTCCTGGGCTGTC			
*PCNA*	F:GAACCTCACCAGCATGTCCA	86	58	NM_001034494
	R:ACGTGTCCGCGTTATCTTCA			
*GAPDH*	F:CCACCACATACTCAGCACC	244	56	NC_037332
	R:AGTTCAACGGCACAGTCAA			
*MYOG*	F:GCGCAGACTCAAGAAGGTGA	126	58	NM_001111325
	R:TGCAGGCGCTCTATGTACTG			
*MYH1*	F:GGCCAGACTGTAGAGCAGGTAT	84	54	NM_174117
	R:GGCAACCATCCACAGGAACATC			
*MYOD1*	F:GTGCAAACGCAAGACGACTA	128	56	NM_001040478
	R:GCTGGTTTGGGTTGCTAGAC			
*MYF5*	F:AGACGCCTGAAGAAGGTCAA	134	56	NM_174116
	R:AGCAGCTCCTGCAGACTCTC			
*MDM4*	F:CACTGCCTACCTCACA	113	50	NM_001046169
	R:ATCCCACTCCTCAAAC			
*TP53*	F:GATGAATGTCCGAATGAAGC	141	54	NM_174201
	R:ACCGTAATTGCCAGGGTA			
*NOP56*	F:GCCTGTCTGCCCTGAT	93	54	NM_001033622
	R:CTGTGGATGCTGGATACTT			
*GAR1*	F:CGGCAGAGGAGGATT	108	50	NM_001035303
	R:CAAGGGTGCAGGAACT			
*DKC1*	F:CAGGCAAAGAGTATGTGGG	122	54	NM_001105395
	R:CGCAGCAATGAGTGGG			
*NPM1*	F:AAACCGTCAACACCAA	140	54	NM_001035441
	R:GGAAGGGAACCACCT			
*UBTF*	F:AACCTCCCATGAATGGTTAT	154	58	XM_002696042
	R:TCAGCCTGCTTCTTGTAGTG			
*NOP58*	F:GTGGCTGATGCTAAACT	187	54	NM_001192957
	R:ACCTGTATCGGGACAA			
*NCL*	F:GTCTTTGGCAATGAAAT	104	58	NM_001206660
	R:GTAACTTTGTAAGGCAGAT			
*FBL*	F:AGGGCGTCTTCATTTGTCG	133	54	NM_001205400
	R:TTCCAGGCACGGTACTCAAT			
*GADD45A*	F:CTGCGTGTCAGCAACCC	197	54	NM_001034247
	R:TGATCCATGTAGCGACTTTCC			
*BAX*	F:ATCGGAGATGAATTGGACAG	95	58	NM_173894
	R:GCCGCCACTCGGAAAA			
*CCNB1*	F:AACTATGCTGGACTACGAT	152	54	NM_001045872
	R:TAACAACGAGAAGGGATT			
*CDKN1A*	F:GCGGTGGAACTTCGACTTTG	99	58	NM_001098958
	R:ACGGGCAGGTAGAGCTTGG			

## Data Availability

The original contributions presented in this study are included in the article. Further inquiries can be directed to the corresponding authors.

## Abbreviations

The following abbreviations are used in this manuscript:

SMSCs	Skeletal muscle satellite cells
RPS27L	Ribosomal protein S27-like
p53/TP53	Tumor protein p53
MDM2	Mouse double minute 2 homolog
MDM4	Mouse double minute 4 homolog
NCL	Nucleolin
DAPI	4′,6-diamidino-2-phenylindole dihydrochloride
MyHC	Myosin heavy chain
MyoD	Myogenic differentiation 1
MyoG	Myogenin
Ki67	Marker of proliferation Ki-67
PCNA	Proliferating cell nuclear antigen
GAPDH	Glyceraldehyde-3-phosphate dehydrogenase
siRNA	Small interfering RNA
RNAi	RNA interference
PBS	Phosphate-buffered saline
TBST	Tris-buffered saline with Tween-20
BSA	Bovine serum albumin
SDS-PAGE	Sodium dodecyl sulfate–polyacrylamide gel electrophoresis
PVDF	Polyvinylidene difluoride
ECL	Enhanced chemiluminescence
HRP	Horseradish peroxidase
qRT-PCR	Quantitative reverse transcription polymerase chain reaction
RNA-seq	RNA sequencing
DEGs	Differentially expressed genes
PCA	Principal component analysis
GSEA	Gene set enrichment analysis
KEGG	Kyoto Encyclopedia of Genes and Genomes
GO	Gene Ontology
SD	Standard deviation
SEM	Standard error of the mean
ANOVA	Analysis of variance
BCA	Bicinchoninic acid assay
DMEM	Dulbecco’s modified Eagle medium
FBS	Fetal bovine serum
IF	Immunofluorescence
WB	Western blot
ActD	Actinomycin D
